# Isolation of Novel Bacterial Strains *Pseudomonas extremaustralis* CSW01 and *Stutzerimonas stutzeri* CSW02 from Sewage Sludge for Paracetamol Biodegradation

**DOI:** 10.3390/microorganisms11010196

**Published:** 2023-01-12

**Authors:** Antonio Vargas-Ordóñez, Inés Aguilar-Romero, Jaime Villaverde, Fernando Madrid, Esmeralda Morillo

**Affiliations:** Institute of Natural Resources and Agrobiology of Seville, Spanish National Research Council (IRNAS-CSIC), 41012 Seville, Spain

**Keywords:** biodegradation, paracetamol degrader, sewage sludge, *Pseudomonas extremaustralis*, *Stutzerimonas stutzeri*

## Abstract

Paracetamol is one of the most used pharmaceuticals worldwide, but due to its widespread use it is detected in various environmental matrices, such as surface and ground waters, sediments, soils or even plants, where it is introduced mainly from the discharge of wastewater and the use of sewage sludge as fertilizer in agriculture. Its accumulation in certain organisms can induce reproductive, neurotoxic or endocrine disorders, being therefore considered an emerging pollutant. This study reports on the isolation, from sewage sludge produced in wastewater treatment plants (WWTPs), of bacterial strains capable of degrading paracetamol. Up to 17 bacterial strains were isolated, but only two of them, identified as *Pseudomonas stutzeri* CSW02 and *Pseudomonas extremaustralis* CSW01, were able to degrade very high concentrations of paracetamol in solution as a sole carbon and energy source, and none of them had been previously described as paracetamol degraders. These bacteria showed the ability to degrade up to 500 mg L^−1^ of paracetamol in only 6 and 4 h, respectively, much quicker than any other paracetamol-degrader strain described in the literature. The two main paracetamol metabolites, 4-aminophenol and hydroquinone, which present high toxicity, were detected during the degradation process, although they disappeared very quickly for paracetamol concentrations up to 500 mg L^−1^. The IC_50_ of paracetamol for the growth of these two isolates was also calculated, indicating that *P. extremaustralis* CSW01 was more tolerant than *S. stutzeri* CSW02 to high concentrations of paracetamol and/or its metabolites in solution, and this is the reason for the much lower paracetamol degradation by *S. stutzeri* CSW02 at 2000–3000 mg L^−1^. These findings indicate that both bacteria are very promising candidates for their use in paracetamol bioremediation in water and sewage sludge.

## 1. Introduction

The term pharmaceuticals (PCs) includes a wide collection of chemical families such as anti-inflammatories, analgesic, antidepressant, antiepileptic, antimicrobial, therapeutic or veterinary drugs used to prevent or treat diseases. In the last few years, an increasing number of studies have detected their presence in surface and groundwater or in soils and, for this reason, PCs are considered as emerging contaminants in the environment [1]. They are considered anthropogenic contaminants whose presence in the environment is due to the continuous discharge of effluents from hospitals, pharmaceutical industries, and, especially, wastewater treatment plants (WWTPs), where PCs are continually discharged into the sewer system through human excreta. Depending on the diversity of the structures in PCs, their hydrophobic or hydrophilic character, and their functional groups, some of them can be completely degraded by the conventional treatment processes in WWTPs and they will not be present in effluent treated water; however, some others, which are highly soluble in water but poorly degraded, or even the metabolites of some degraded PCs, can remain in such effluents. Finally, those PCs or metabolites with poor biodegradability but high hydrophobic character can remain adsorbed in the highly hydrophobic surface of sewage sludge [2,3].

PCs can reach the aquatic environment through the discharge of WWTPs effluents [4], and can reach the soil through irrigation with such effluents and/or by the use of sewage sludge from WWTPs as fertilizers [5]. Furthermore, PCs leaching or runoff from agricultural soils treated with such biosolids are considered potential entry routes into different ecosystems [6]. Therefore, PCs in the environment has become a growing concern due to their potential to impair the ecosystem and/or to induce health risks [7].

Acetaminophen or Paracetamol (APAP, from its chemical name N-acetyl-para-aminophenol) is one of the most used pharmaceuticals worldwide, with analgesic, anti-inflammatory and antipyretic properties, and with an increasing consumption every year [8]. APAP is considered safe and it is available without a prescription; however, due to its widespread use, it is detected in various environmental matrices such as surface and ground waters, sludge, sediments, soils or even plants [9]; and for this reason, it is considered an emerging pollutant [10]. Although the concentration of APAP in these matrices is considered very low, negative effects and toxicity on different species have been described due to its presence; being of special importance is its accumulation in certain aquatic organisms, in which it can induce reproductive, neurotoxic or endocrine disorders [11,12,13]. Bouissou-Schurtz et al. [14] indicated the ecotoxicity potential of APAP with a risk quotient (RQ) in France of 1.6, and Fekadu et al. [15] observed a RQ value of 43 in developing countries.

Due to the high variety of matrices where APAP is dispersed, it is necessary to decrease its environmental impact, especially through the reduction of its concentration in WWTPs effluents, which is the major route for its entry into the environment. For this purpose, some physicochemical techniques have been developed for the treatment of wastewaters, such as those that use electrochemical advanced oxidation processes, which used to be highly efficient [16,17], but they are all linked to high operational costs due to the drastic reactions conditions needed. On the contrary, a more environmentally friendly treatment, and a relatively cheap one, is the use of microorganisms able to degrade APAP through bioaugmentation processes. In the last twenty years there has been a variety of bacteria studied that have the capacity to use APAP as a carbon and energy source, contributing to its biodegradation. Most of them belong to the genus *Pseudomonas* although not all bacteria from this genus were equally effective [18,19,20,21]. Palma et al. [22] used consortia formed by bacterial communities obtained from sludge from WWTPs to observe which genus had a more increasing abundance through the biodegradation process. They observed that 50 mg/L of APAP were degraded in 48 h, and that a strain from the genus *Pseudomonas* was the most abundant in the degrader consortium, although the genera *Flavobacterium*, *Dokdonella* and *Methylophilus* also increased in abundance, suggesting they have a role in APAP degradation.

Bacterial strains from the genus *Pseudomonas* were able to degrade much higher APAP concentrations (some of them above 2000–3000 mg/L), but other bacterial strains have also demonstrated their ability to degrade APAP, most of them isolated from sewage and activated sludge. Wei et al. [23] used the strain *Cupriavidus necator* F1; Zhang et al. [20] found, for the first time, that *Stenotrophomonas* sp. F1 could degrade this drug; Park and Oh [24] used *Ensifer* sp. POKHU; and Chen et al. [25] degraded APAP with *Shinella* sp. HZA2. Chopra and Kumar [26] isolated five bacterial strains from sewage samples that were able to degrade APAP: *Staphylococcus sciuri* DPP1, *Bacillus subtilis* DPP3, *Bacillus paralicheniformis* DKP1, *Enterococcus faecium* DKP2, and *Enterococcus faecium* DDP2.

In this study, new bacterial strains capable of metabolizing and degrading APAP as a sole source of carbon and energy were isolated from sewage sludge. The phylogenetic relationship among all the isolates were studied and two of them, not previously described as APAP degraders, were selected as the most active; able to degrade high concentrations of APAP in a shorter time than other strains previously described in the literature. Increasing amounts of APAP were used in the biodegradation processes to observe the effectivity of the two selected strains in a wide range of concentrations in solution. Moreover, the two main metabolic derivatives generated were determined throughout the degradation process in order to relate their absence with the detoxification and bioremediation potential of APAP and its metabolites in water.

## 2. Materials and Methods

### 2.1. Chemicals and Materials

Analytical standards of APAP (C_8_H_9_NO_2_, N-(4-hidroxifenil)etanamida (IUPAC)), also known as paracetamol (CAS 103-90-2, purity ≥ 99%), 4-aminophenol (4-AP) (CAS 123-30-8, purity > 98%) and hydroquinone (HQ) (CAS 123-31-9, purity > 99%) were purchased from Sigma-Aldrich (Madrid, Spain). Their molecular structures and some of their properties are shown in Table 1. Sewage sludge was obtained from a Wastewater Treatment Plant (WWTP) in the city of Seville (southwest Spain) that processes part of the wastewater generated in the city as well as that arriving from a hospital. The most relevant physicochemical properties of the sludge used are described in Table 2.

### 2.2. Enrichment Cultures and Bacterial Identification

APAP-degrader microbial consortia were isolated from approximately 10 g of the sludge described above through an enrichment culture using APAP (200 mg L^−1^) as a sole carbon and energy source, together with 50 mL of Mineral Salt Medium (MSM) (sterilised by autoclave Auster-G, P-Selecta with one cycle at 121 °C, inlet pressure of 103 kPa, for 20 min). MSM contained principal and essential trace elements (g L^−1^): 0.5 K_2_HPO_4_; 0.5 KH_2_PO_4_; 0.01 NaCl; 0.2 MgCl_2_ ·6H_2_O; 0.02 CaCl_2_; 0.0004 ZnSO_4_ ·7H_2_O; 0.0004 CoCl_2_ ·6H_2_O; 0.0003 MnSO_4_; 0.01 of ethylenediaminetetraacetic acid (EDTA) and 0.0003 (NH_4_)_6_Mo_7_O_24_ ·4H_2_O and 1 (NH_4_)_2_HPO_4_ (GPR RECTAPUR^®^, VWR). Cultures were incubated at a constant temperature of 30 ± 1 °C in an orbital shaker at 180 rpm. Every week, 5 mL of the previous culture were transferred to a new flask with a fresh solution of the same sterile mineral medium MSM spiked with 200 mg L^−1^ APAP and incubated again. This process was repeated for 7 weeks. After the seventh enrichment transfer, an aliquot of the culture was plated simultaneously on Luria-Bertani (LB)-Agar medium (25 g L^−1^ LB Broth and 20 g L^−1^ agar) and MSM-APAP plates (200 mg L^−1^) and incubated for 3 days at 30 ± 1 °C. The LB broth contained (g L^−1^): 10.0 tryptone, 5.0 yeast extract, and 10.0 NaCl (BD Difco™, Fisher Scientific). The consortium obtained was mixed with a 40% glycerol solution (50/50 V/V), stored in Eppendorf microtubes and kept at −80 °C.

Different APAP-degrader bacterial strains were isolated from the consortium on LB agar medium petri dishes based on their morphological characteristics (size, surface, colour, margin, shape, elevation, etc.). Identification of those strains that showed in-plate growth capacity was achieved by extraction of the total genomic DNA from the pure culture using a GeneJET Genomic DNA Purification Kit (Thermo Scientific, Waltham, MA, USA) and via amplification of their 16S rRNA genes by PCR, using universal oligonucleotide primers 27F (5′-AGAGTTTGATCCTGGCTCAG-3′) and 1492R (5′-GGTTACCTTGTTACGACTT-3′), and posterior sequencing at the STABVIDA laboratory (Caparica, Portugal) via Sanger sequencing where, in addition to primers 27F and 1492R, primers 518F (5′-CCAGCAGCCGCGGTAATACG-3′) and 800R (5′-TACCAGGGTATCTAATCC-3′) were used. A total of 5 bacterial strains were identified. Taxonomic identification was carried out by comparison with the NCBI database (National Centre for Biotechnology Information, Bethesda, MD, USA) using the BLASTn algorithm [28,29]. The bacterial 16S DNA sequence of these strains were submitted to the GenBank database and deposited with their corresponding accession numbers.

A phylogenetic analysis was performed to accurately determine the taxonomic position of the strains. To determine the phylogenetic affiliation, a multiple sequence alignment of the 16SrRNA genes was performed using the ClustalW algorithm with default settings inside MEGA11 software [30]. Phylogenetic trees based on the 16SrRNA genes were constructed using the maximum-likelihood [31], neighbor joining [32] and maximum-parsimony algorithms in MEGA11. The tree robustness was evaluated using a boot-strap analysis of 1000 resamplings [33].

### 2.3. Inoculum Preparation

Bacterial strains of two isolates (CSW01 and CSW02) were cultivated in LB medium in the presence of APAP (200 mg L^−1^) (150 rpm, 30 ± 1 °C) and harvested through centrifugation (7000 rpm, 10 min) at the beginning of the stationary phase. The pellets obtained were washed twice in MSM solution in order to remove completely the remains of the LB medium and APAP previously added, and then suspended in MSM solution. Bacterial growth was monitored by optical density (OD_600 nm_) on a VWR UV-3100 spectrophotometer and by colony-forming units (CFU) of serial dilutions on LB medium plates. The initial cell density of each strain added in the degradation experiments was approximately 10^8^ CFU mL^−1^ [34].

### 2.4. Inhibitory Concentration of APAP for Bacterial Growth

The half maximal inhibitory concentration (IC_50_) for bacterial growth is the concentration of APAP that causes a 50% drop in bacterial growth. This value was estimated from duplicate batch cultures in LB medium under aerobic conditions with different APAP concentrations (10, 50, 250, 500, 2000 and 5000 mg L^−1^) and without APAP (as a reference for normal bacterial growth) during an incubation period of 24 h at 150 rpm and 30 ± 1 °C. The bacterial growth for IC_50_ determination was analyzed by counting the number of CFUs/mL present on each culture after the incubation time. Based on these values, the percentage of bacterial viability (CFUs/mL in a culture with a drug concentration over the CFUs/mL in the culture without drug) was calculated for the different tested APAP concentrations. The IC_50_ values were estimated using Microsoft Excel.

### 2.5. Biodegradation Assays

Biodegradation experiments of APAP in solution in a monosubstrate system were performed in triplicate by inoculating the isolated bacterial strains. All microcosm components were sterilised before the assays. In total, 50 mL of MSM sterile solution spiked with APAP (10, 200, 500, 2000 or 3000 mg L^−1^) were added to 250 mL Erlenmeyer flasks. Although the control flasks remained without inoculation (in order to observe a possible abiotic degradation), the sample flasks were inoculated to obtain 10^8^ CFU mL^−1^ of the corresponding bacterial strain. These solutions were incubated on a temperature-controlled rotary shaker at 150 rpm and 30 ± 1 °C and protected from light. At the beginning of the experiment and at periodic intervals, 1 mL samples were taken from the flasks to 1.5 mL Eppendorf microcentrifuge tubes in order to determine the APAP residues remaining in solution. To ensure the recovery of APAP present in the supernatant and accumulated in the microbial biomass, the samples were subjected to a process of three consecutive cycles of freezing and thawing, in order to break the cell walls. Afterwards, the samples were centrifuged at 11,000 rpm for 2 min. The supernatant was then measured by High Performance Liquid Chromatography (HPLC) as described below.

### 2.6. Models of Biodegradation Kinetics

An Excel file provided by the FOCUS [35] workgroup on degradation kinetics and the Solver tool (Microsoft statistical package) were used to fit all APAP biodegradation curves to the best possible kinetic model. The parameters were optimized by adapting the recommendations by FOCUS to our biodegradation processes, using the least-squares method. The biodegradation curves were fitted to the kinetic simple first-order model (SFO) or a first-order multicompartment model (FOMC) using the following equations:

M_t_ = M_0_ · e^−kt^   (SFO)

DT_50_ = ln 2/k   (SFO)

DT_90_ = ln 10/k   (SFO)

M_t_ = M_0_/((t/β) + 1)^α^   (FOMC)

DT_50_ = β (2 (1/α) − 1)   (FOMC)

DT_90_ = β (10 (1/α) − 1)   (FOMC)

α = 1/(N − 1)

β = (M_0_/1 − N)/[k(N − 1)]

where M_t_ and M_0_ are the concentrations of the remaining APAP (mg L^−1^) at time t and immediately after spiking the solution, respectively, and k is the rate constant of degradation (day^−1^). For the FOMC model, α corresponds with the shape parameter determined by the coefficient of variation of k values, and β is the location parameter. Meanwhile, DT_50_ refers to the time required for the pollutant concentration to reach half of its initial value. These models were chosen for their simplicity, as well as their potential to adjust the measured dissipation kinetic datasets for monophasic biodegradation. The Chi-square (χ^2^) test was performed to determine which model fitted best by considering chi-squared and scaled error values with a least square 0.05 to estimate the appropriateness of the model and to assess the accuracy of each resulting fit.

### 2.7. Detection of APAP and Its Main Metabolites

APAP concentration was determined by HPLC coupled to a UV-vis detector (LC-2010AHT, Shimadzu) using a Liquid Purple C-18 column (4 × 150 mm; Análisis Vínicos, Spain) as the stationary phase. The mobile phases consisted of a mixture of methanol and water (10:90 *v*/*v*) at pH adjusted to 3 using orthophosphoric acid 85%. Flow was set at 1.2 mL min^−1^, the injection volume was 25 µL, and the detection wavelength was 244 nm. APAP concentration in the sample vials was determined by a comparison with the calibration curves. Additionally, to determine the concentration of the APAP metabolites produced during the degradation process, 4-AP and HQ, HPLC coupled to a fluorescence detector (Shimadzu RF-10APXL, Kioto, Japan) with a C-18 column (4 × 150 mm) was employed. The mobile phase contained a phosphate buffer (9 g of KH_2_PO_4_ and 0,063 g of K_2_HPO_4_ in 1 L of ultrapure water) and methanol (80/20 *v*/*v*) with excitation and emission wavelengths at 290 and 355 nm, respectively. Flow was set at 1 mL min^−1^ and the injection volume was 5 µL.

## 3. Results and Discussion

### 3.1. Enrichment Cultures for the Isolation and Identification of APAP-Degrading Bacterial Strains

Aiming to obtain bacteria able to resist, as well as degrade, high concentrations of APAP, enrichment cultures were initiated with the pharmaceutical as the only carbon and energy source using a sewage sludge sample as the inoculum. The concentration used (200 mg L^−1^) was higher than those found in nature or even in WWTPs. However, it was chosen to impose a selective pressure on the bacterial community to allow the enrichment and isolation of bacterial strains with the ability to degrade this compound. Other authors have followed a similar approach [25,36]. A total of 17 bacteria with different morphological characteristics were isolated, 5 of which showed some growth capacity in minimal medium plates containing APAP. It should be noted that the expected growth of bacteria in this type of medium is often much milder than in plates of rich medium.

A 16S rRNA gene sequencing analysis of these five bacteria was performed. Their sequence was then compared and submitted to the GenBank database, thus characterizing these isolates as *Pseudomonas extremaustralis*, *Stutzerimonas stutzeri*, *Pseudomonas nitroreducens*, *Pseudomonas citronellolis and Brevundimonas olei.* The similarities amongst the sequences of our isolates and that of the other strains present in the GenBank database is shown in Table 3. The 16S rRNA sequences of all five isolates are available on GenBank with their accession numbers OP729928 (*Pseudomonas extremaustralis* CSW01), OP729929 (*Stutzerimonas stutzeri* CSW02), OP727816.1 (*Pseudomonas nitroreducens* CSW03), OP727817.1 (*Pseudomonas citronellolis* CSW04), and OP684288.1 (*Brevundimonas olei* AP2-7.2).

**Figure 1:**
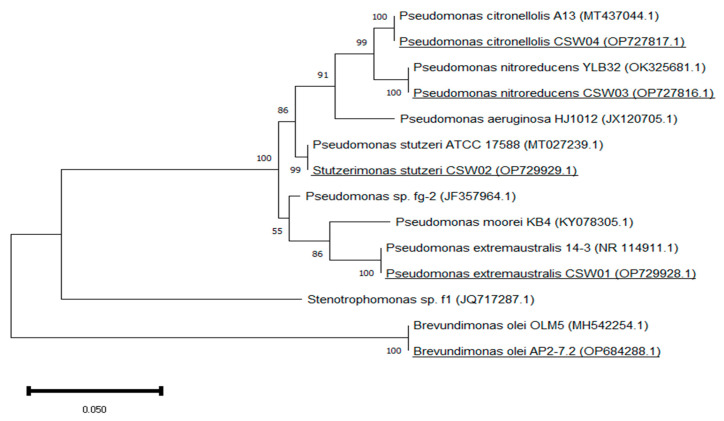
Maximum Likelihood tree based on 16S rRNA genes showing the relationships between the strains isolated from the enrichment culture (underlined) using other strains of the same species as a reference. Bootstrap values are expressed as percentages of 1000 replicates. The same branches representation was recovered by the neighbor-joining and maximum-parsimony algorithms. The scale bar indicates 0.050 substitutions per nucleotide position in the 16S rRNA gene sequences tree.

The phylogenetic affiliation of our isolates amongst themselves and with a selection of strains previously described as APAP degraders is shown in Figure 1: *P. aeruginosa* HJ1012 [19], *Pseudomonas* sp. fg-2 [20], *P. moorei* KB4 [21] and *Stenotrophomonas* sp. f1 [20]. As it can be observed, all isolates were different and had significant divergence as none of them were present in a single clad. This tool graphically shows how the enrichment process has boosted the development of some bacteria over others. All of the isolates that have been identified were included in the Gram-negative bacteria group; four out of five of them being part of the *Pseudomonas/Stutzerimonas* genus. This is consistent with the findings of Palma et al. [22], who monitored the proportion of bacteria with a role in the degradation of APAP present in enrichment culture on MSM medium inoculated with two types of sludge, and noticed bacteria from the *Pseudomonas* genus to have a dramatically high relative abundance in just 6 days.

The isolate CSW02, identified as *S. stutzeri,* is remarkably closely related with bacteria from the *Pseudomonas* genus (Figure 1), although this specie tends to be represented on a clad of its own. The reason is that, while historically this specie was classified as part of the *Pseudomonas* genus, most recently it has been withdrawn from this group by taxonomists. Studying more meticulously the phylogenetic affiliations of the *stutzeri* specie with the rest of *Pseudomonas*, specifically *Pseudomonas aeruginosa* as it is the type species of the genus, Laculat et al. [37] and Gomila et al. [38] created the new genus *Stutzerimonas*, where *P. stutzeri* and another nine former species of Pseudomonas were transferred. Because of the novelty nature of this classification, the two names are still used as synonyms. The phylogenetic tree also represents how closely related are some of our isolates with other strains previously described as APAP degraders. Especially, *P. extremaustralis* CSW01 and *S. stutzeri* CSW02 are proximately related to the degraders *Pseudomonas moorei* KB4 and *Pseudomonas* sp. fg-2. This could suggest a shared metabolic pathway in the case of APAP biodegradation.

### 3.2. APAP Degradation in Aqueous Solution

**Figure 2:**
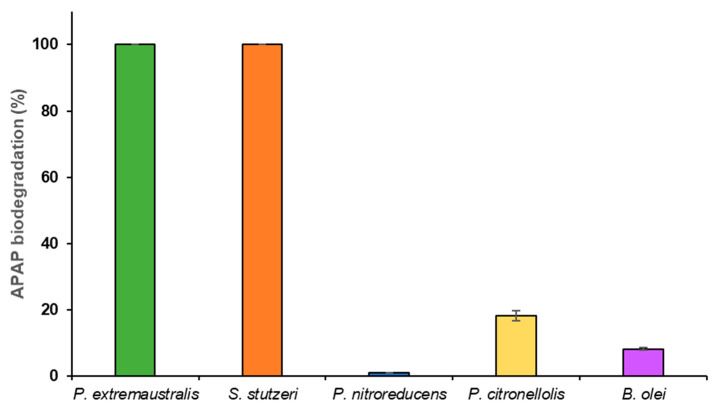
APAP biodegradation in solution by different bacterial strains in the presence of 10 mg L^−1^ of the drug after 24 h of incubation.

All five isolates that showed the ability to grow in plates of MSM-APAP were tested to see it they were able to degrade the pharmaceutical in solution. For this purpose, a quick screening was performed with all of them, aiming to quantify their degradation capacity after a 24 h exposure in MSM supplemented with 10 mg L^−1^ of APAP. *Pseudomonas extremaustralis* CSW01 and *Stutzerimonas stutzeri* CSW02 showed the highest degradation capacity, with 100% of the APAP supplied being degraded after 24 h. They were followed by *Pseudomonas citronellolis* CSW04, with an 18% degradation, and *Brevundimonas olei* AP2-7.2, with an 8% degradation of the pollutant. The degradation capacity *of Pseudomonas nitroreducens* CSW03 was negligible (Figure 2).

**Figure 3:**
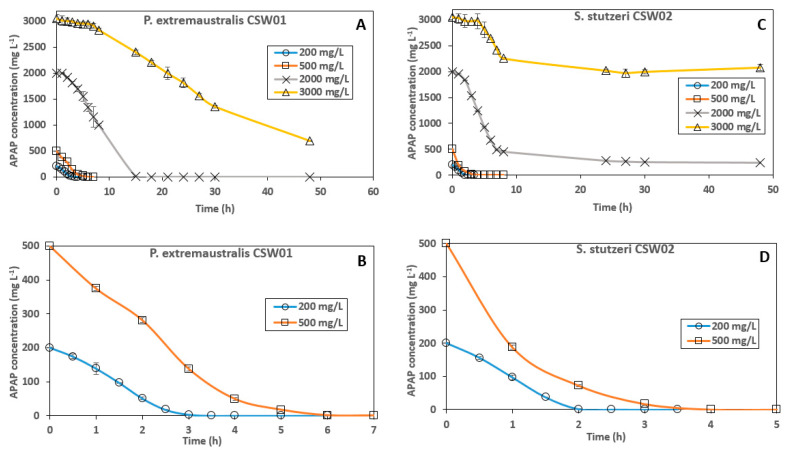
Influence of different initial concentrations of APAP in the degradation capacity of *P. extremaustralis* CSW01 (**A**,**B**) and *S. stutzeri* CSW02 (**C**,**D**). ((**B**,**D**) show enlarged details of (**A**,**C**), respectively).

In the light of these results, *Pseudomonas extremaustralis* CSW01 and *Stutzerimonas stutzeri* CSW02 were selected as the most promising candidates and kinetics curves of their biodegradation processes were built using higher APAP concentrations (Figure 3).

These two bacterial strains are also considered low pathogenic (those included in risk group 1 based on the WHO Laboratory Biosafety Manual [39], which is an important aspect to take into account because those considered to be virulent have to be avoided for bioaugmentation and bioremediation purposes.

While other bacteria included in the *Pseudomonas* genus have already been described as APAP degraders, such as *P. moorei* KB4 [21] or *P. aeruginosa* HJ1012 [19], no strains of *Pseudomonas extremaustralis* or *Stutzerimonas stutzeri* have previously been described, according to our knowledge, as APAP degraders. However, several strains of *S. stutzeri* (named as *P. stutzeri*) have been reported to degrade other organic pollutants, such as alkyl derivatives of aromatic hydrocarbons [40], crude oil [41], textile dye [42] and other pharmaceuticals, such as alprazolam [43]. *Pseudomonas extremaustralis*’s discovery is much more recent [44]. It is more famously known for its extremophile characteristics and its ability to produce polihydroxybutyrates, so very few works have focused on its use for biodegradation, until now, although it is capable of degrading diesel [45]. It is also, as far as we know, the first time that a member of this species has been isolated from sewage sludge. It has previously been isolated from Antarctic ponds [44], soil samples [46] and permafrost sediments [47], and it has been detected in activated sludge by Ji et al. [48] using Illumina Miseq sequencing, yet not isolated.

Figure 3 shows the influence of different initial concentrations of APAP in the degradation capacity of *P. extremaustralis* CSW01 and *S. stutzeri* CSW02. Five different concentrations were measured, but data from 10 mg L^−1^ could not be presented in the figure because the APAP disappeared in the first minutes after inoculation with both strains. Biodegradation curves of the control samples remained stable throughout the length of the experiment (not shown); therefore, the degrading abiotic processes can be considered negligible. Lag phases were not perceived for 200 and 500 mg L^−1^ on either of the strains, as the residual APAP concentration sharply decreased. Nevertheless, a small lag phase can be observed while testing higher concentrations, becoming longer as the concentration increased. The kinetic parameters obtained from APAP biodegradation by these two bacterial strains are shown in Table 4. In terms of degradation efficiency, *S. stutzeri* CSW02 showed a faster removal rate than *P. extremaustralis* CSW01 for the two lower concentrations tested (following SFO kinetics for both strains), as evidenced by their k, DT_50_ and DT_90_ values (Table 4). However, this tendency changes as the concentration rises. In assays with 2000 mg L^−1^, which followed a FOMC kinetic, *S. stutzeri* CSW02 still biodegraded APAP faster than *P. extremaustralis* CSW01, with a DT_50_ of 4.2 h versus 6.0 h, although *P. extremaustralis* was able to reach a 100% degradation of the pharmaceutical, while *S. stutzeri* was not (Figure 3). In the case of an initial concentration of 3000 mg L^−1^, *P. extremaustralis* CSW01, even though it suffered from a more significant lag phase than *S. stutzeri* CSW02, showed a better biodegradation performance, succeeding in removing 50% of the initial concentration in just 25.4 h, and reaching an extent of APAP biodegradation of about 80% in 48 h. On the contrary, *S. stutzeri* showed a DT_50_ value of 60.8 h, with an extent of biodegradation of only 33% (Table 4, Figure 3).

These results, observed for very high APAP concentrations, also correlate positively with those of other isolated degrader strains tested. *P. aeruginosa* HJ1012 was able to degrade 2200 mg L^−1^ in 42 h, but could not degrade 100% of an initial APAP concentration of 2500 mg L^−1^ (Hu et al., 2013). *Pseudomonas* sp. fg-2 and *Pseudomonas* sp. f2 [20] degraded 2000 and 2500 mg L^−1^, respectively, in around 48 h, though they reached saturation at 2500 and 3000 mg L^−1^. Similar results were obtained by [25] with the Gram-negative bacterium *Shinella* HZA2, which was able to degrade 2000 mg L^−1^ in 40 h, but reached an extent of saturation of approximately 90% for 2500 mg L^−1^ and 50% for 3000 mg L^−1^.

However, when we focus on a lower concentration, such as 500 mg L^−1^ (which is still very high), to compare the two strains under study with other APAP-degraders, it can be observed that the degradation kinetic to reach complete APAP degradation is much quicker with our strains than with others described in the literature (Table 5).

**Figure 4:**
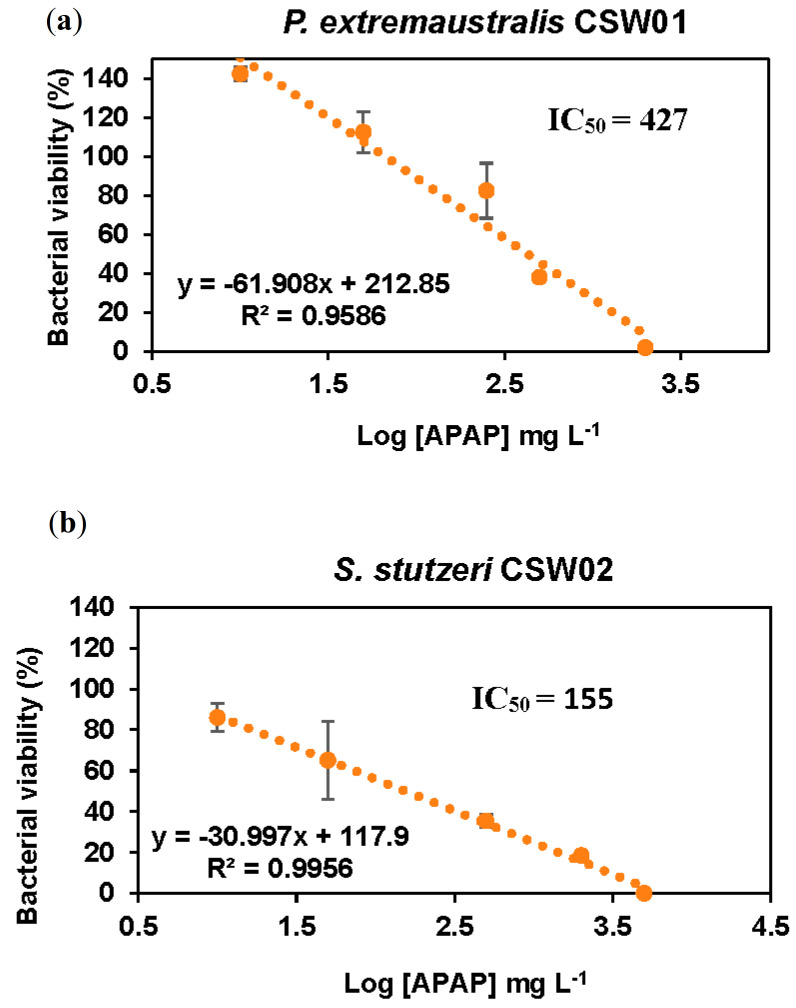
Graphical representation of the best fitting linear curves for bacterial viability as a function of the logarithm of APAP concentration for *P. extremaustralis* CSW01 (**A**) and *S. stutzeri* CSW02 (**B**).

In order to explain this behavior, and also the lower APAP degradation observed in Figure 3 for *S. stutzeri* in comparison *P. extremaustralis* at the highest concentrations, estimations of the APAP IC_50_ for both strains were carried out. This test was performed via the comparison of the CFUs mL^−1^ growth after 24 h of incubation in cultures with and without the pharmaceutical. The bacterial viability as a function of the logarithm of APAP concentration fits reasonably for both strains as a linear tendency curve (Figure 4). IC_50_ gave a value of 427 mg L^−1^ for *P. extremaustralis CSW01* and 155 mg L^−1^ for *S. stutzeri CSW02*. The higher IC_50_ value of *P. extremaustralis* shows that this degrading strain is more tolerant to higher concentrations of APAP and/or its metabolites in solution. This is in agreement with its increased capacity to degrade APAP at concentrations of 2000 and 3000 mg L^−1^ in comparison to *S. stutzeri,* and correlates with the kinetic results obtained (Table 4). Similarly, *P. extremaustralis,* at low concentrations of APAP (10 mg L^−1^), shows a surprisingly high bacterial viability (Figure 4), which again shows its ability to resist in the presence of APAP and/or possible metabolites formed.

### 3.3. Detection and Quantification of Main Metabolites

In order to observe the production of possible APAP metabolites and to enlighten the metabolic pathway that followed the degradation of the parent compound, HPLC analysis with fluorescence detection was performed on the samples corresponding to the degradation curve with an initial concentration of 500 mg L^−1^ of APAP. 4-aminophenol (4-AP) and hydroquinone (HQ) were detected, simultaneously, using the same analytical conditions (previously described in Section 2.5).

**Figure 5:**
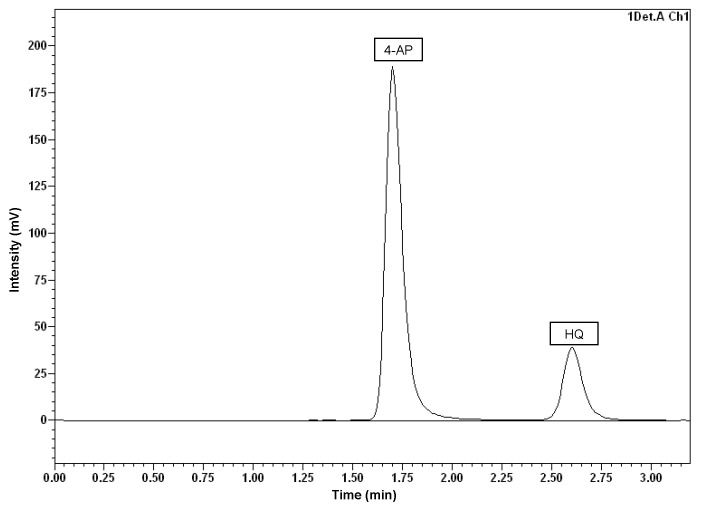
Chromatogram obtained by HPLC analysis illustrating the detection of the APAP biodegradation intermediates: 4-aminophenol (4-AP) and hydroquinone (HQ).

**Figure 6:**
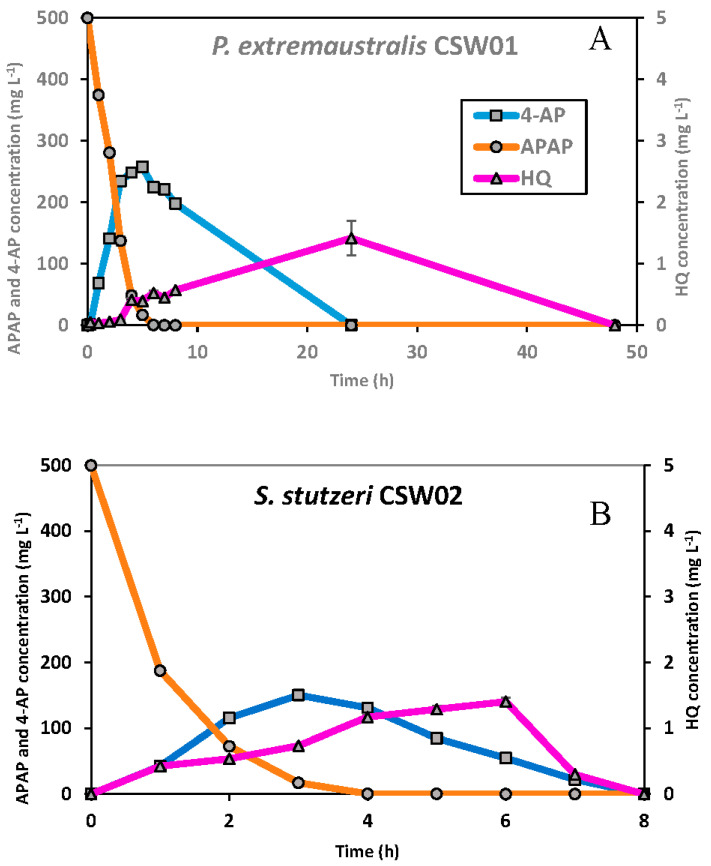
Biodegradation curve for APAP (○), 4-AP (□), and HQ (∆) in solution, with an initial APAP concentration of 500 mg L^−1^ for *P. extremaustralis* CSW01 (**A**) and *S. stutzeri* CSW02 (**B**).

As can be seen in Figure 5, 4-AP appeared at 1.6 min, whereas HQ did so at 2.7 min. In the experiment with *P. extremaustralis* CSW01, 4-AP was detected right at the beginning of the degradation process, reaching its maximum measured concentration (257 mg L^−1^) after 5 h, and decreasing to non-detectable levels in 24 h. The HQ concentration started rising significantly 2 h after the beginning of the assay, reaching its maximum (1.4 mg L^−1^) at 24 h and diminishing to a non-detectable concentration after 48 h (Figure 6A).

Alternatively, *S. stutzeri* CSW02, which is capable of degrading APAP faster than *P. extremaustralis* CSW01 at the lower concentrations studied (200, 500 and 2000 mg L^−1^), showed an ability to metabolize both by-products in less time. 4-AP reached its maximum concentration of 150 mg L^−1^ three hours after the beginning of the experiment, while HQ reached its maximum after 6 h with 1.4 mg L^−1^. Both metabolites had disappeared from the solution in only 8 h (Figure 6B).

The disappearance of both metabolites during the biodegradation of APAP suggests that both degrading strains would be able to eliminate environmental toxicity, which is mainly caused by at least one of the metabolites studied as 4-AP, which has already been shown to be more toxic than APAP itself. Zur et al. [21] reported the toxicity of APAP and its key metabolite formed during microbial degradation, 4-AP, using the Microtox test with a bioluminescence *Vibrio fischeri* (*Allivibrio fischeri*) strain. The authors confirmed that the intermediates formed during the microbial degradation of APAP are characterized by a significantly higher toxicity than the parent compound. The fact that both metabolites disappeared relatively quickly when the initial concentration was 500 mg L^−1^ gave, as a result, the rapid decrease in toxicity, not affecting the kinetics of the degradation process. On the contrary, these metabolites would appear at very high levels when the APAPs initial concentration in solution was 2000–3000 mg L^−1^, increasing the toxicity and slowing down the degradation rate; even stopping it in the case of *S. stutzeri*, which showed a lower tolerance to toxicity, as indicated by its corresponding IC_50_.

## 4. Conclusions

Of the seventeen bacterial strains isolated from sewage sludge using APAP as the only carbon and energy source, five of them showed growth capacity, and four of these being part of the Pseudomonas/Stutzerimonas genus. Pseudomonas extremaustralis CSW01 and Stutzerimonas stutzeri CSW02 showed the highest degradation capacity, being described for the first time as APAP-degraders. Moreover, the species Pseudomonas extremaustralis was also reported for the first time to be isolated from sewage sludge.

*S. stutzeri* CSW02 showed a faster removal rate than *P. extremaustralis* CSW01 for APAP concentrations lower than 2000 mg L^−1^; however, this tendency changed as the concentration increased. This is due to the fact that *P. extremaustralis* CSW01 is more tolerant than *S. stutzeri* CSW02 to higher concentrations of APAP and/or its metabolites in solution.

Two APAP toxic metabolites, 4-aminophenol and hydroquinone, were detected through the biodegradation process. At lower APAP concentrations, both metabolites disappeared relatively quickly, decreasing the toxicity and not affecting the biodegradation process. At higher concentrations, these toxic metabolites appeared at very high levels, increasing the toxicity and greatly slowing down (or even stopping) the degradation rate. Nevertheless, with both of these strains, APAP degradation at about 500 mg L^−1^ is much quicker than with any other APAP-degrader strain described previously in the literature.

## Figures and Tables

**Table 1:** Formulas, molecular structures and some properties of the compounds discussed in this work [27].

Name	IUPAC Name	Structure	Molecular Weight (g mol^−1^)	Water Solubility (20 °C)	pKa	Log K_OW_
Acetaminophen; paracetamol	N-(4-hydroxyphenyl)acetamide	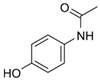	151.16	14 g L^−1^	9.38	0.46
4-aminophenol	4-aminophenol	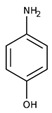	109.13	16 g L^−1^	pK1 = 5.48 pK2 = 10.46	0.04
Hydroquinone	Benzene-1,4-diol	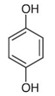	110.11	6.7 g L^−1^	9.96	0.59

**Table 2:** Physico-chemical properties of the sewage sludge sample (mean of four replicates). Standard deviations are given in parenthesis.

Organic matter (%)	48.0	(7.2)
pH	8.25	(0.03)
Nitrogen (Kjeldahl) (%)	5.05	(0.76)
P_2_O_5_ (%)	6.00	(1.20)
K_2_O (%)	0.42	(0.07)
CaO (%)	7.11	(1.42)
MgO (%)	0.94	(0.17)
Fe (mg kg^−1^)	>50,000	(8000)
Cd (mg kg^−1^)	<2	(0.3)
Cu (mg kg^−1^)	203	(30)
Ni (mg kg^−1^)	17	(2)
Pb (mg kg^−1^)	37	(7)
Zn (mg kg^−1^)	620	(105)
Hg (mg kg^−1^)	0.40	(0.10)
Cr (mg kg^−1^)	32	(6)

**Table 3:** Phylogenetic affiliations of bacterial strains isolated from acetaminophen enrichment cultures from sewage sludge.

Strain (Accession Number)	NCBI Affiliation (Accession Number)	Similarity	Class; Order; Family; Genus
**CSW1** **(OP729928.1)**	Pseudomonas extremaustralis 14-3 (NR_114911.1)	100%	Gammaproteobacteria; Pseudomonadales; Pseudomonadaceae; Pseudomonas
**CSW2 (OP729929.1)**	Pseudomonas stutzeri ATCC 17588 (MT027239.1)	100%	Gammaproteobacteria; Pseudomonadales; Pseudomonadaceae; Stutzerimonas
**CSW3 (OP727816.1)**	Pseudomonas nitroreducens YLB32 (OK325681.1)	100%	Gammaproteobacteria; Pseudomonadales; Pseudomonadaceae; Pseudomonas
**CSW4 (OP727817.1)**	Pseudomonas citronellolis A13 (MT437044.1)	100%	Gammaproteobacteria; Pseudomonadales; Pseudomonadaceae; Pseudomonas
**AP2-7.2 (OP684288.1)**	Brevundimonas olei OLM5 (MH542254.1)	100%	Alphaproteobacteria; Caulobacterales; Caulobacteraceae; Brevundimonas

**Table 4:** Kinetic parameters calculated from the APAP biodegradation curves in solution after inoculation with *P. extremaustralis* CSW01 and *S. stutzeri* CSW02.

Bacterial Strain	APAP Concentration (mg L^−1^)	Model Kinetic	K (Day^−1^)	α (Day^−1^)	β (Day^−1^)	DT_50_ (h)	DT_90_ (h)	χ^2^ *	Scaled Error	R^2^
*Pseudomonas extremaustralis* CSW01	200	SFO	0.684	-	-	1.01	3.37	12.59	16.3	0.933
	500	SFO	0.448	-	-	1.55	5.14	15.51	27.3	0.962
	2000	FOMC	-	151,924	1,318,434	6.02	20.0	22.36	168	0.878
	3000	FOMC	-	36,266	1,326,513	25.3	84.2	18.31	127	0.964
*Stutzerimonas stutzeri* CSW02	200	SFO	1.022	-	-	0.68	2.25	12.59	14.1	0.952
	500	SFO	0.995	-	-	0.70	2.31	12.59	3.79	0.999
	2000	FOMC	-	218,321	1,313,894	4.17	13.9	18.31	160	0.912
	3000	FOMC	-	15,129	1,327,039	60.8	202	18.31	176	0.748

* χ^2^ calculated values ≤ χ^2^ corresponding to the tabulated value (*p* ≤ 0.05).

**Table 5:** Degradation of APAP by different bacterial strains under similar concentrations.

Strain	Initial Concentration (mg L^−1^)	Complete Degradation (h)	Reference
*Stutzerimonas stutzeri* CSW02	500	4	This study
*Pseudomonas extremaustralis* CSW01	500	6	This study
*Pseudomonas* sp. ST1	777	72	[49]
*Cupriavidus necator* F1	400	48	[23]
*Pseudomonas aeruginosa* HJ1012	500	12	[19]
*Stenotrophomonas* sp. f1	400	116	[20]
*Pseudomonas* sp. fg-2	600	23	[20]
*Pseudomonas* sp. f2	600	38	[20]
*Ensifer* sp. POKHU	400	65	[24]
*Shinella* sp. HZA2	600	30	[25]

## Data Availability

Not applicable.
